# Definition and evolution of right ventricular dysfunction in critically ill COVID-19 patients. Author’s reply

**DOI:** 10.1186/s13613-022-01056-y

**Published:** 2022-08-30

**Authors:** Michelle S. Chew, Saga Jansson, Meriam Åström Aneq, Jan Engvall

**Affiliations:** 1 Department of Anaesthesiology and Intensive Care, Biomedical and Clinical Sciences, Linköping University, Linköping, Sweden; 2 Department of Clinical Physiology and Department of Health, Medicine and Caring Sciences, Linköping University, Linköping, Sweden

**Keywords:** COVID-19, Intensive care, Acute myocardial injury, Ventricular dysfunction, Echocardiography, Cardiac troponins

We thank Dr. Chotalia and coworkers for their interest in our paper [1]. We fully agree that there remains a gap in knowledge regarding how the right ventricle is affected by COVID and that this may only be elucidated in large, prospective studies with serial echocardiographic examinations.

The lack of a statistical association between right ventricular systolic dysfunction (RVD) and mortality does not imply a lack of a true finding. As discussed in our study, there may be several reasons for the lack of this association, including a strict definition of RVD and the absence of a selection bias. Notably, all 13 patients with RVD suffered an acute myocardial injury (AMInj) compared to 76% of those without RVD (*p* = 0.055). Similarly, among patients with AMInj, 28% had RVD compared to none of the patients without AMInj. HsTnT concentrations were higher in those with RVD, a finding that is congruent with previous studies. Therefore, we believe that these findings have possible clinical significance.

Another finding was the higher incidence of AMInj in patients with LVD (32% (with LVD) vs. 11% (without LVD); *p* = ns) and higher peak hsTnT values (126 ng/l (with LVD) vs. 43 ng/l (without LVD); *p* = 0.006). Therefore, the possible importance of left ventricular systolic dysfunction should not be discounted yet.

An important aspect to our study is the finding of the acuity of myocardial injury in this critically ill population. A majority of patients (59%) in this population already suffered from myocardial injury on ICU admission. We further demonstrated that acute injury occurred during critical care and investigated the relationship between early (within 72 h of admission) echocardiographic findings and this acute injury, something that is rarely studied. A logical next question is what causes acute injury in a heart that is already compromised, and can this be preventable? Can echocardiography help identify these patients at an earlier stage? Therefore, we disagree with the author’s contention that it is unsurprising that RVD did not associate with AMInj and that an association would have been observed if TTE had been performed at the time of the injury. Since we do not know when this injury will appear, the relationship between RVD and AMInj can only be studied with serial and comprehensive echocardiography with simultaneous cardiac troponin measurements, a study that is currently in progress in our group.

We observed (contrary to our expectations) an increased ICU mortality of 20% among those without RVD, compared to 8% among those with RVD. This did not reach statistical significance, and as discussed in our paper, we would caution against making any robust conclusions with these small numbers (8% = 1 death!).

Regarding the definition of RVD: we arbitrarily chose our definition and have reported our reasons for doing so. We disagree with Chotalia et al. that there are validated definitions of RVD by the ESICM. They refer to an excellent ‘Letter’; however, that publication does not provide a definition for RVD and even explicitly states that there is no consensus definition for right ventricular failure [2]. We also emphasize that there is a difference between right ventricular systolic dysfunction and right ventricular failure. There are several approaches to investigate right heart systolic dysfunction and failure described in previous literature; based on systolic function parameters [3], the presence of acute cor pulmonale [4] and based on the association between RV dilatation and elevated CVP [5]. We were guided by the PRICES statement and acknowledged that different definitions may have led to different results. One limitation of our study was the absence of documentation of septal dyskinesia that is required for the definition of acute cor pulmonale (ACP).

We were surprised by our results and while there may be several reasons for our divergent findings, and we believe that the effect of selection and reporting bias is an important consideration when assessing previous studies and planning future studies. LVD seems to occur as frequently as RVD and should not be disregarded. AMInj occurs even against a background of already ongoing injury at ICU admission. Clinical outcomes such as the need for vasopressors/inotropes and mechanical ventilation may be more relevant than mortality. The best way to assess right ventricular dysfunction/failure in ARDS is the subject of ongoing research. In fact, this is the goal of the RECHOicu group that is currently being formed (pers. Comm. Prof. Antoine Vieillard-Baron). Until then, we are only able to transparently report our methodology, results, and offer possible reasons for our findings. To conclude otherwise based on the current results would be nefarious and unscientific.

## Data Availability

The data from the original study will be made available after publication, upon application to the corresponding author and within the terms of the Global Data Protection Regulation and the Swedish Patient Data Law (2008:355). To avoid the possibility of identifying individual cases, detailed data are not given in the paper but may be requested from the corresponding author.
